# Markers of lower respiratory tract infections in emergency departments

**DOI:** 10.1186/2049-6958-8-20

**Published:** 2013-03-15

**Authors:** Dursun Tatar, Gunes Senol, Ceyda Anar, Gultekin Tibet

**Affiliations:** 1 Department of Respiratory Medicine, Dr. Suat Seren Chest Diseases and Surgery Training and Research Hospital, Izmir, Turkey; 2 Department of Infectious Diseases and Clinical Microbiology, Dr. Suat Seren Chest Diseases and Surgery Training and Research Hospital, Yenisehir, Izmir, Turkey

**Keywords:** C-reactive protein, Gram stain, Lower respiratory tract infections, Sputum culture, White blood cells

## Abstract

**Background:**

Acute respiratory tract infections are the common causes for admission to emergency department. Appropriate diagnosis and initiating treatment on time are important for reducing morbidity and mortality rate due to lower respiratory tract infection (LRTI). The aim of this study is to determine if C-reactive protein (CRP) levels and white blood cells (WBC) count are considerable markers to help rapid diagnosis and prediction of antibiotic need for lower respiratory infections in emergency departments. The relationships between infectious agents and those markers have also been evaluated.

**Methods:**

Study subjects and control group were selected by defined criteria. Patients were analyzed and assessed for CRP and WBC, sputum Gram stain and culture besides routine laboratory tests and chest X-Rays.

**Results:**

One hundred and ninety four episodes out of 175 patients were evaluated for the study. CRP level and WBC count were detected significantly higher in patients ofstudy group than in those of control group. *Pseudomonas aeruginosa* and *Haemophilus influenzae* were the pathogens most often isolated.

**Conclusion:**

In conclusion, CRP and WBC sputum are important markers for diagnosis of LRTI at the emergency departments and results of microbiological analysis of respiratory specimens were correlated with these markers.

**Trial registration:**

Registation number of ethic committee of Dr. Suat Seren Chest Diseases and Surgery Training and Research Hospital: 28.04.2006/114

## Background

Respiratory tract infections are among the most common reasons for admission to emergency departments (ED) [1]. Although antibiotic treatment is considered necessary only for community-acquired pneumonia and small subgroups of the other lower and upper respiratory tract infections, antibiotics are prescribed to about 80% of patients consulting for lower respiratory tract infection (LRTI) [2]. Signs and symptoms are of limited value in identifying those patients who need antibiotic treatment [1]. Diagnostic uncertainty and patient-related factors, such as patient expectations and pressure, often lead to unjustified antibiotic prescriptions by emergency physicians [3].

LRTI mainly include bronchitis, bronchiolitis and pneumonia [4]. Acute exacerbations of chronic obstructive pulmonary disease (AE-COPD) and pneumonia often cause LRTIs in ED visits [5]. Emergency departments are required to diagnose and manage patients with severe pneumonia associated with a high morbidity and mortality and antibiotic timing is accepted as a measure of hospital quality [6,7].

COPD was detected in 23% of respiratory diseases and caused mortality in 3.3% of all hospitalizations in Turkey [8]. In developed countries, it is the fourth leading cause of death with 4.3%; but it is the second with 10.8% rate in developing countries [9].

The Gram stain method is one of the most commonly used technique for the rapid diagnosis of bacterial infections. The result of the Gram stain can be reported in less than 10 minutes, giving the physician a reasonable idea of its identity upon which to base treatment. Gram staining provides a presumptive diagnosis and some indication of the organism involved without waiting for culture results. The specimen is obtained by deep cough and should be grossly purulent. Culture should be performed only if the specimen meets strict cytological criteria (except for detection of legionella or mycobacteria). Controversy surrounds the role of Gram stain and culture analysis of expectorated sputum in patients with community-acquired pneumonia. Most reports suggest that these tests have poor positive and negative predictive value in LRTI patients [10]. However, these tests should be performed to try to identify etiologic organisms in the hope of reducing microbial resistance to drugs, unnecessary drug costs, and avoidable side effects of empirical antibiotic therapy [11].

In Turkey, according to national statistics, 14.8% of all hospitalizations are due to respiratory disease other than tuberculosis. Lower respiratory infections except tuberculosis are the cause of the whole mortality in 4.1% percentage. Respiratory diseases and respiratory infections are reported 13.8% and 3.3% rate, respectively [12].

The aim of this study is to determine etiological bacterial agents and the possible inflammatory markers in patients with or without adequate respiratory secretions who presented to the ED of one hospital with symptoms of respiratory infection.

## Methods

The study was planned as a prospective, open observational study in ED of a tertiary referral center for chest diseases and chest surgery.

Patients who met the inclusion criteria in 2006 were enrolled in the study.

Inclusion criteria:

1. Acute illness present for 14 days or less;

2. At least one lower respiratory tract symptom (cough, sputum production, dyspnea, wheezing, chest pain);

3. No alternative explanation of cough for example, not sinusitis, pharyngitis, or a new presentation of asthma.

4. And/or fever.

### Exclusion criteria

Confirmed tuberculosis cases were excluded from the study. Patients affected with cardiovascular diseases, thromboembolism, pregnancy, those who used corticosteroids within the last six months, and those who had been discharged from hospital within seven days before were not included.

Control cases were randomly selected from patients with respiratory complaints, retrospectively.

**Figure 1:**
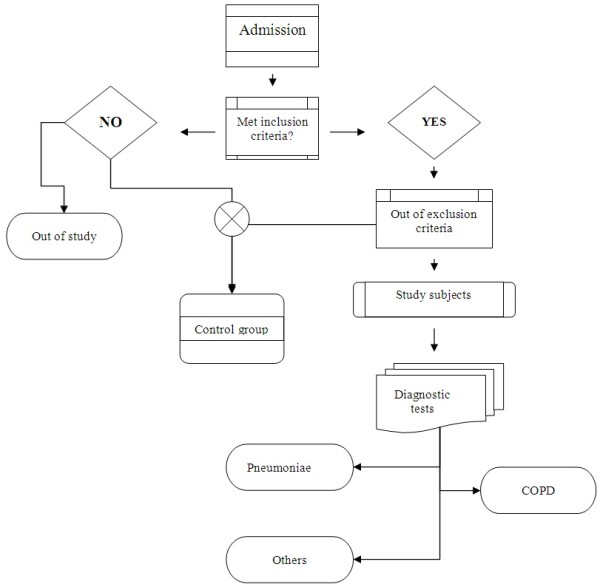
Flowchart of the selection method of study and control groups.

Diagnosis of COPD was confirmed by spirometry after acute exacerbation in patients at their first admission to the hospital. Pneumonia was diagnosed by infiltration on chest X-Ray in addition of inclusion criteria. Flow chart of the selection method is shown in Figure 1.

#### Microbiology

Bacteriological sputum cultures, WBC count and CRP test were performed for all cases besides other conventional tests according to the nature of cases on the day of admission. Sputum samples were sent to the bacteriology division. They were previously examined macroscopically for evaluating the qualification for culture by laboratory technician. Samples which included saliva or food within insufficient respiratory secretions were rejected. Sputum samples were analyzed by Gram stains and culture methods. Sputum purulence was identified as above 25 polymorphonuclear leucocytes (PNL) per low power microscopic field [11]. Identification of micro-organisms was done by conventional microbiological methods and semi-automated systems (Crystal, BD, Maryland, USA). Antibiotic susceptibility of bacteria was done by disc diffusion method according to CLSI recommendations. CRP and WBC were studied in serology division of microbiology laboratory. CRP levels were tested by nephelometric method. (Array 360 System, Beckman Coulter, USA). WBC was counted by Coulter hematology analyzer (Beckman Coulter, USA).

SPSS 15 program was used for statistical analysis. Distribution of data was evaluated by Kolmogorov Smirnov test. Analysis of parametric variables was performed with t test; Kruskal Wallis and Mann-Whitney tests were applied for nonparametric distributed variables. Chi-square test was used for analyzing categorical data. *p* under 0.05 was accepted as significant.

This research was carried out in compliance with the Helsinki Declaration, and approved by ethic committee of Dr. Suat Seren Chest Diseases and Surgery Training and Research Hospital, İzmir, Turkey with 28.04.2006/114 registration number.

## Results

In the study period, 31,257 episodes of 21,346 patients were examined and 2,298 cases of pneumonia and other lower respiratory infections were detected in 1,480 in patients in ED.

The cases which met the inclusion criteria (194 episodes of 175 patients) and 50 controls were studied. The pathological conditions of control patients were twenty one common cold, ten pharyngitis, eight asthma, four pneumothorax, two thoracic injury, two foreign body aspiration, one corpulmonare, one acute laryngitis and one sinusitis.

Table 1 shows demographic data of the cases and controls.

**Table 1:** Demographic data of the patients and controls

	**n**	**Gender**	**Age**	**Smoking**	
				**%**	**Package/year**
AECOPD	115	109 M, 6 F	64.4 ±11.4	94%	52.7 ± 24.7
**PNEUMONIA**	64	55 M, 9 F	64.3 ± 12.4	74%	51.6 ± 26.0
*OTHERS	15	10 M, 5 F	68.3 ± 9.6	66%	54.5 ± 25.2
**TOTAL CASES**	194	174 M, 20 F	64.2 ± 11.7	92%	52.5 ± 25.6
**CONTROLS**	50	31 M, 19 F	46.2 ± 17.4	42%	20.5 ± 8.5

* Asthma n = 3, interstitial pulmonary diseases n = 2, acute bronchitis, tracheitis, silicosis, operated esophageal carcinoma.

*F*, Female, *M*, Male.

Prevalence of smoking and pack/year were found significantly higher in the LRTI group than in controls.

**Figure 2:**
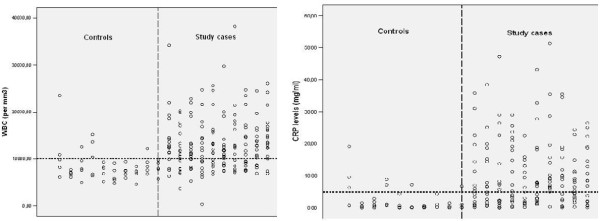
**a. WBC values of study and control cases. b**. CRP values of study and control cases.

Symptoms and laboratory findings of the patients are reported in Tables 2 and 3, respectively. Shortness of breath was the major symptom in all patients in the LRTI group. Fever was seen in higher rate in pneumonia cases than in other cases. Comparative values of CRP and WBC in cases and controls are shown in Figure 2a and 2b. CRP and WBC values were significantly higher in LRTI subjects than in controls (p < 0.001). Differential leukocyte count indicated normal or neutrophilic increase in patients.

**Table 2:** Symptoms of the patients

	**N**	**SYMPTOMS**					
		**n (%)**					
		**Dyspnea**	**Cough**	**Expectoration**	**Fever**	**Chest pain**	**Hemoptysis**
AECOPD	115	115 (100)	65 (56.5)	52 (45.2)	1 (0.9)	1 (0.9)	2 (1.7)
**PNEUMONIA**	64	59 (92.1)	44 (68.7)	37 (57.8)	15 (23.4)	2 (3.1)	5 (7.8)
**OTHERS**	15	13 (86.6)	7 (46.6)	4 (26.6)	1 (6.6)	0	3 (20)

**Table 3:** Laboratory findings of study subjects and controls

	**N**	**CRP**	**WBC**
AECOPD	115	9.8 + 9.9	12640 + 4800
**PNEUMONIA**	64	11.2 + 8.9	14490 +6400
**OTHERS**	15	14.0 + 12.5	13100 + 6200
**CONTROLS**	50	3,5 ± 1.9	8100 ± 3100

**Figure 3:**
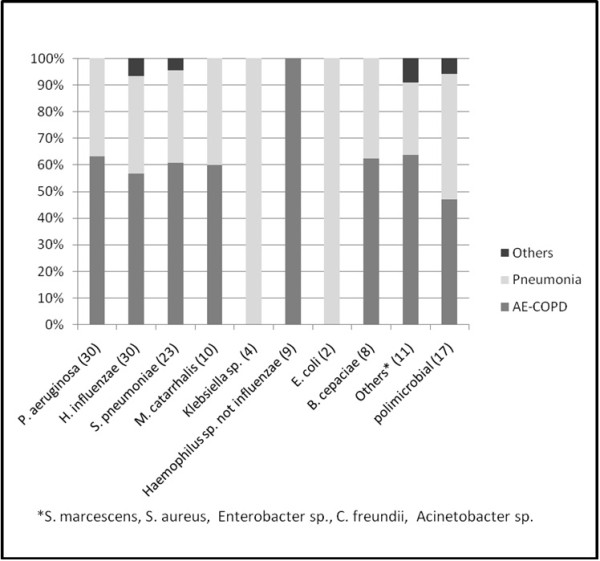
Bacterial agents isolated in sputum samples according to study groups.

In Figure 3, microbiological analysis of sputum samples are shown. WBC and CRP levels according to microscopic features of sputum are detailed in Table 4. The results showed that when pathogen isolation was not possible, CRP was an important marker for diagnosis of LRTI. *Pseudomonas aeruginosa* and *Haemophilus influenzae* were the most frequent pathogens.

**Table 4:** WBC and CRP levels according to microscopic features of sputum

		**>5 mg/dl**	**0.8-5 mg/dl**	**<0.8 mg/dl**	**WBC >10000/ml**
		***n (mean ± SD)***			
Microscopy* of sputum samples	**>25 PNL**	20 (17,7 ± 12,3)	7 (2,4 ± 1,1)	1 (0,3 ± 0)	19 (12275 ± 5300)
	**10-25 PNL**	10 (13,2 ± 7,2)	2 (2,8 ± 0,4)	0	8 (15680 ± 8390)
	**<10 PNL**	14 (18,8 ± 16,7)	5 (2,9 + 0,9)	8 (0,3 ± 0,2)	19 (13640 + 6000)
**No expectoration**		2 (29,2 ± 16,4)	6 (2,9 ± 1,2)	3 (0,5 ± 0,4)	5 (9950 ± 4440)

*per low power field (×10).

Mean number of previous hospital admissions was significantly higher in *P. aeruginosa* infections (p = 0.142). In infections due to other agents, the numbers of previous admissions did not show significant difference.

## Discussion

We have reported in this study serum CRP responses, WBC count and sputum purulence in patients with lower respiratory infections according to data obtained from a population-based study that includes all spectra of the disease together with strict criteria for unequivocal diagnosis. The present results provide evidence for the usefulness of the CRP assay, WBC count and sputum microbiology in the diagnosis of LRTI in ED and to evaluate the need for antibiotic therapy.

CRP is an acute-phase protein with levels quickly rising during inflammatory processes [2]. Relationship between CRP and infection of the lower respiratory tract was indicated by different studies in literature. High CRP levels were reported sensitive and specific to determine lower respiratory diseases [13]. Almirres *et al*. reported that CRP levels have been shown to be also useful in confirming the diagnosis, since they were significantly higher in patients with true pneumonia than in those in whom the diagnosis was not confirmed at follow up [14]. Macfarlane *et al*. in a study of lower respiratory tract infections in patients attending an outpatient setting, reported that 65% of patients with radiographically confirmed disease showed high serum CRP levels (above 5 mg/dL) compared with 40% in those with radiographic findings that were consistent with infection, and 11% in those who had no changes consistent with infection [1]. Melbye *et al.* showed that serum CRP level of above 5 mg/dL in patients with symptoms of respiratory infection who had been treated as outpatients had a sensitivity of 54% and a specificity of 95% for the diagnosis of pneumonia [15]. In a study by Castro-Guardiola *et al.* on cases of pneumonia diagnosed at the hospital ED, mean serum CRP levels of 18.1 mg/dL were found in cases of confirmed pneumonia and much lower titers in false-positive cases [16]. Gomperts *et al.* showed that purulent exacerbations were associated with significant bronchial and systemic inflammation and positive bacterial cultures. CRP values were reported significantly high in the purulent bronchitis [17]. A CRP test result adds incremental information to the physicians’ information obtained from medical history and physical examination [18]. The introduction of CRP testing to assist antibiotic prescribing decisions in LRTI resulted in a reduction of antibiotic use [2].

High WBC count was found associated with AECOPD as well as with pneumonia in literature. Sin *et al*. pointed out that blood WBC were elevated in a directly proportional manner with severity of exacerbation [19]. Gan *et al*. also reported high WBC in AECOPD with has been showing with increased systemic inflammation that reflected the severity of exacerbation [20]. Roche *et al.* concluded that leukocyte count would be remarkable in patients hospitalized for COPD exacerbations with purulent sputum [21]. Besides, some studies disagreed that WBC elevates in COPD, because it was found a normal WBC count and markedly elevated CRP level in AECOPD [13,22]. Thus, it has been suggested that it is reasonable to check CRP level in addition to WBC count in patients admitted the ED due to fever.

Performing microbiological research as early as during ED admission could help a definitive diagnosis. Atasever *et al*. suggested that sputum samples, which were collected from patients at the moment they applied to ED, are important for isolation of etiologic agents of lower respiratory infections. They also reported that high CRP level and high WBC count were found significant in diagnosis of respiratory infections. They concluded that results would help when deciding if antibiotics should initially be used to treat patients with AECOPD [23].

On the other hand, van der Meer reported in his review that testing for CRP is not sufficiently sensitive and specific to rule out an infiltrate on chest radiograph and bacterial etiology of LRTIs. He concluded that the evidence was not sufficient to support a wide introduction of CRP as a rapid test to guide antibiotics prescription [24].

Additionally we found that isolation of *P. aeruginosa* was getting higher along with high number of previous admissions. Russel *et al*. reported that a quarter of potentially pathogenic microorganisms colonize the patients with COPD during their stable periods. Exacerbation is associated with the overgrowth of these microorganisms and with the appearance of *P. aeruginosa* in the lower airway, which is associated with exacerbation symptoms independent of load [25]. Ohmagari et al proposed that extended spectrum antibiotics may need to be used as first-line empirical therapy for COPD with prior infection that requires hospitalization, and as alternative, antipseudomonal antibiotic regimens may need to be considered [26].

Although it has not been tested in our study, procalcitonin (PCT) is an important biomarker worth to discuss in this issue. PCT was found very useful to assess the severity of respiratory disease and the necessity of antibiotic use as CRP in various studies, and it has been reported that PCT is a more sensitive marker than CRP. Cut off values of PCT were recommended between 0,15 ng/ml and 0,5 ng/ml for the best sensitivity and specificity in different studies [27,28].

A limitation of this study could be considered that data were not available about prior antibiotic consumption of patients that might have an effect on likelihood of respiratory flora and sputum culture. Other limitations of the study were the inability of identification of viral agents and the lack of studying PCT.

## Conclusion

In conclusion we beleive that CRP and WBC are good diagnostic markers especially for patients who do not expectorate sputum sample. Microbiological investigation of sputum samples should be done in adequate cases in order establish a rational antibiotic treatment and avoid unnecessary drug resistance. History of hospital admission is important for selection of empiric antibiotics.

## Competing interests

The authors affirm that they have no conflict of interest in relation to the subject of the study.
